# Fatal myocarditis-associated *Bartonella quintana* endocarditis: a case report

**DOI:** 10.4076/1752-1947-3-7325

**Published:** 2009-07-17

**Authors:** Ambroise Montcriol, Fréderic Benard, Florence Fenollar, Alberto Ribeiri, Marc Bonnet, Fréderic Collart, Catherine Guidon

**Affiliations:** 1 Department of Anesthesia and Intensive Care Unit, Hopital La Timone, rue Saint Pierre, 13885 Marseille Cedex 5, France; 2 Unité des rickettsies, Université de la Méditerranée, rue Saint Pierre, 13885 Marseille Cedex 5, France; 3 Department of Cardiac Surgery, Hopital La Timone, rue Saint Pierre, 13885 Marseille Cedex 5, France

## Abstract

**Introduction:**

*Bartonella* spp. infection is not rare and must be considered with great care in patients with suspected infective endocarditis, particularly if regular blood cultures remain sterile. Management of these infections requires knowledge of the identification and treatment of these bacteria.

**Case presentation:**

A 50-year-old Senegalese man was admitted to our Department of Cardiac Surgery with a culture-negative endocarditis. Despite valvular surgery and adequate antibiotic treatment, recurrence of the endocarditis was observed on the prosthetic mitral valve. Heart failure required circulatory support. Weaning off the circulatory support could not be attempted owing to the absence of heart recovery. Bacteriological diagnosis of *Bartonella quintana* endocarditis was performed by molecular methods retrospectively after the death of the patient.

**Conclusions:**

This case report underlines the severity and difficulty of the diagnosis of *Bartonella quintana* endocarditis. The clinical picture suggested possible *Bartonella quintana* associated myocarditis, a feature that should be considered in new cases.

## Introduction

*Bartonella quintana* is a Gram-negative bacterium first identified as the cause of louse-borne epidemic trench fever in Europe during World War One. Additional manifestations of *B. quintana* infection including endocarditis, bacillary angiomatosis, chronic bacteremia and pericarditis have been reported [1]. *B. quintana* infection is an important cause of culture-negative endocarditis [2]. We report a patient with *B. quintana* endocarditis followed by a myocardial dysfunction which may evoke fulminant myocarditis.

## Case presentation

A 50-year-old Senegalese man was hospitalized for a 6-month history of dyspnea, fever and 7 kg weight loss. His past medical history was significant for a myocardial infarction 4 years earlier. No valvulopathy was reported. He testified to good living conditions in Senegal and denied homelessness and substantial alcohol use.

**Figure 1:**
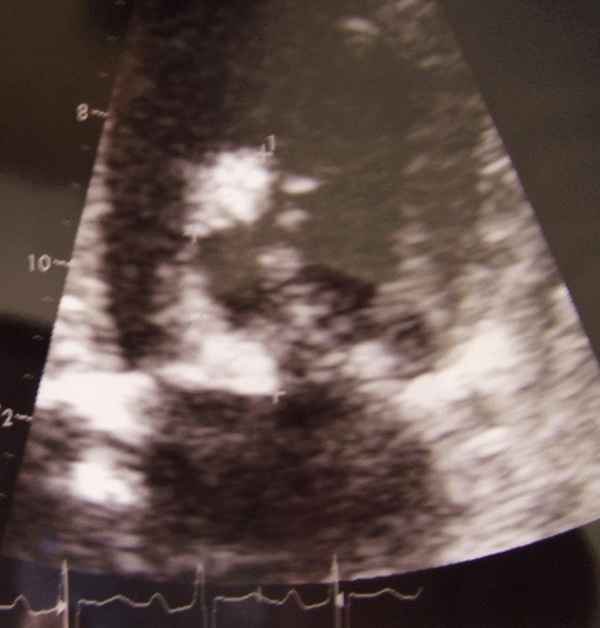
**Pre-operative transthoracic echocardiography showing mitral vegetation and mitral chordal rupture**.

Transthoracic echocardiography revealed major aortic and mitral regurgitations, aortic and mitral vegetation, a mitral chordal rupture, systolic pulmonary arterial pressure (SPAP) at 60 mmHg and a left ventricle ejection fraction (LVEF) of 45% with apical and septal akinesia (Figure 1). A coronary angiogram revealed no significant stenosis except for a left circumflex artery occlusion. Pre-operative tomodensitometry showed two disseminated septic emboli in the left cerebral cortex and in the left kidney. Initial blood cultures remained negative. Thus, an empiric antibiotic treatment with amoxicillin (12 g daily intravenously) and gentamicin (200 mg daily intravenously) was started. Aminoglycoside peak concentration was monitored daily and the dose adjusted. The hemodynamic pattern of the patient required bioprosthetic aortic and mitral valve replacement with a cardiopulmonary bypass 3 days after his admission.

On initial admission to the surgical intensive care unit, his hemodynamic status was stable with blood pressure at 130/60 mmHg with epinephrine 0.8 mg/hour and dobutamine 12 µg/kg/minute. Weaning from the mechanical ventilation was successful at the 6th postoperative hour. Progressively, a right ventricle failure appeared (right ventricle hypokinesia, SPAP of 45 mmHg at echocardiography) with renal and hepatic dysfunction (oliguria, blood urea nitrogen level of 10 mmol/L, creatinine level of 183 µmol/L, alanine aminotransferase (ALAT) level of 20 IU/L, aspartate aminotransferase (ASAT) level of 309 IU/L, bilirubin level of 40 mg/dL) and a troponin Ic level of 159 IU/L on the second postoperative day. Thereafter, cardiogenic shock was observed. Serial cardiac enzymes were consistent with major myocardial injury (troponin Ic >500 IU/L). Right heart catheterization confirmed myocardial dysfunction (pulmonary artery occlusion pressure 25 mmHg, cardiac index 1.5 L/min/m^2^). Thus, extracorporeal membrane oxygenation (ECMO) with a cardio-pulmonary bypass was set up as a bridge to heart recovery. Despite circulatory support, inhaled nitric oxide and continuous veno-venous hemodiafiltration, hemodynamic status remained precarious (blood pressure at 90/40 mmHg with epinephrine 3.5 mg/hour and dobutamine 10 µg/kg/minute). Echocardiography showed a LVEF of 35%, low left and right filling pressures, and good function of the prosthetic valves without any regurgitation.

**Figure 2:**
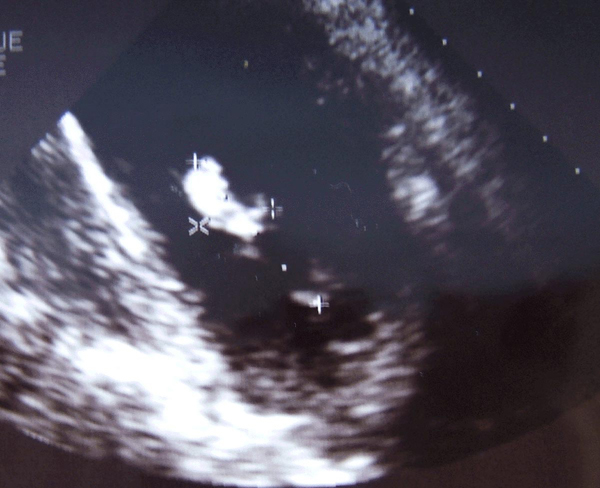
**Transthoracic echocardiography showing large vegetations attached to the prosthetic mitral valve**.

During the following days, no heart recovery was observed. On the fourth day, hepatic function dramatically worsened with severe hypoglycemia, international normalized ratio of 0.18, ALAT level of 1440 IU/L, ASAT level of 2574 IU/L and a bilirubin level of 394 mg/dL. Serial transthoracic echocardiography showed a decrease in the LVEF at 25% and development of large vegetations attached to the prosthetic mitral valve (Figure 2). The patient died of refractory cardiac arrest on the 10th day.

During these 10 days, all blood cultures remained negative. Microbiologic examination of the excised valves showed no organism on Gram stain and no bacteriological growth. Microimmunofluorescence serologies performed on two samples of serum drawn on day 1 and on day 8 remained negative for *Bartonella* spp. (Immunoglobulin G titer <1:100), *Coxiella* spp., *Chlamydia* spp., *Mycoplasma* spp., *Legionella* spp. and *Bosea vestrii*. The Western immunoblotting profile suggested a *Bartonella* spp. endocarditis. On the mitral resected cardiac valve, a broad range polymerase chain reaction (PCR) targeting the 16S ribosomal RNA gene identified a *Bartonella quintana* endocarditis and species-specific PCR amplification targeting the 16S-23S ribosomal DNA intergenic spacer region gene of *Bartonella quintana* confirmed this diagnosis. Thirty days after admission to the surgical intensive care unit, *Bartonella quintana* grew in a blood culture. However, those results were obtained after the death of the patient. Permission for an autopsy was refused.

## Discussion

In developed countries, *B. quintana* endocarditis is confined to specific groups, for example, body-louse infected, alcoholic, homeless and HIV-infected individuals. In France, *Bartonella* spp. account for 3% to 4% of infective endocarditis [3]. A north to south gradient in the distribution of this infection has been reported. Africa is still considered to be endemic for *B. quintana* infections [4,5]. Our patient was Senegalese and lived in Dakar but had no other known risk factors for *B. quintana* infections.

Most reported cases of *B. quintana* endocarditis have affected native valves. Few cases of prosthetic valve endocarditis have been reported [6]-[8]. We report the first case of native valve endocarditis followed by immediate recurrence on a bioprosthetic valve despite adequate antibiotic treatment. We did not observe rapid progression of valvular stenosis as has been described but the rapid development of large vegetation [7].

The cardiac failure and troponin level that we observed were consistent with major myocardial dysfunction. This dysfunction was probably not a complication of the cardiopulmonary bypass usually observed no more than several hours after the surgery. Furthermore, vasopressor requirement was very low in the first postoperative hours. The patient's hemodynamic profile did not suggest septic shock. Thus, we assumed that this dysfunction was due to a direct infection of the myocardium as can be seen in fulminant myocarditis. *Bartonella henselae* myocarditis affecting humans has been reported [9] and a direct infection of myocardial tissue due to *B. quintana* was suspected in a case report [1]. However, in our patient, a biopsy of the myocardium or post-mortem examination was not carried out. Myocarditis could not be confirmed. If *B. quintana* myocardial infection could be proved, it would argue in favor of an aggressive treatment of *B. quintana* induced cardiac failure associating inotropic support and circulatory support until heart recovery.

As we observed, bacteriological diagnosis is difficult and often retrospective. Indeed *Bartonella* spp. are fastidious growing bacteria. Blood or resected valve cultures remain negative for at least 10 days. In the case of blood culture-negative endocarditis, the first screening must include at least *Bartonella* spp. and *C. burnetii* serologies. Usually microimmunofluorescence serology has a high predictive value for the diagnosis of *Bartonella* spp. endocarditis if a cut-off value of 1:800 for *Bartonella* spp. Immunoglobulin G titer is used [10]. In very few cases, as in this report, this technique fails to achieve a diagnosis [10]. Western immunoblotting is usually more sensitive than microimmunofluorescence and allows a specific *Bartonella* species diagnosis when coupled with cross-adsorption [11]. In our patient, the western immunoblotting profile suggested a *Bartonella* spp. endocarditis. Cross-adsorption was not realized because this technique required a large amount of antigens and the species identification was already established by two different PCR assays performed on the cardiac valve sample. In the case of negative serologies, broad-range PCR followed by sequencing performed on cardiac valve samples could help in the etiological diagnosis as this molecular technique allows the detection of numerous bacterial agents. In the case of positive broad-range PCR, species specific PCR should be applied to confirm the diagnosis. The usefulness of molecular techniques in blood is still debated. But their development would be helpful in guiding initial therapy in patients who do not undergo emergency valvular surgery [2].

*B. quintana* endocarditis treatment is non-specific. As in our patient, the delay in diagnosis may provoke serious valve destruction requiring its replacement. More than 90% of patients with *B. quintana* endocarditis require cardiac surgery [12]. *Bartonella* spp. are susceptible *in vitro* to a wide range of agents including penicillins, cephalosporins, aminoglycosides, chloramphenicol, tetracyclines, macrolides, rifampicin, fluoroquinolones and cotrimoxazole. However, aminoglycosides are currently the only known antibiotic class bactericide for *Bartonella* spp. [13]. Patients treated with aminoglycosides for more than 14 days are more likely to survive than those under therapy for a shorter duration [12]. That is the reason why culture-negative endocarditis treatment should associate α-lactamins for 6 weeks and gentamicin intravenously for 2 weeks [14]. In this case report, the choice of ceftriaxone, recommended by the American Heart Association, would probably not have changed the prognosis because it has no more bactericidal activity on *Bartonella* spp. than amoxicillin [15]. If *Bartonella* spp. endocarditis had been suspected, doxycycline could have been added [13]. Given the organ dysfunctions, the persistent sepsis and the disseminated septic emboli, a heart transplant was rejected. This patient died of cardiac failure. Despite adequate antibiotic treatment and valvular surgery, the mortality rate of *B. quintana* endocarditis remains about 12% [12].

## Conclusion

*Bartonella* spp. infection is not rare and must be considered with great care in the case of patients with suspected infective endocarditis, particularly if regular blood cultures remain sterile despite absence of antibiotic treatment. African origin should be considered by itself as a risk factor for *Bartonella* infection. Owing to the high death rate and the difficult diagnosis, culture-negative endocarditis should include aminoglycoside therapy, the only antibiotic treatment bactericide for *Bartonella* spp., for at least 14 days.

The cardiac failure we observed may evoke fulminant myocarditis. Myocardial biopsy could be decisive to determine whether *Bartonella quintana* should be included among the myocarditis infectious agents.

## Consent

Written informed consent was obtained from the patient's next-of-kin for publication of this case report and any accompanying images. A copy of the written consent is available for review by the Editor-in-Chief of this journal.

## Competing interests

The authors declare that they have no competing interests.

## Authors' contributions

AM and FB wrote the manuscript while FF performed the bacteriological identification. AR and FC performed the surgical valves replacements and have revised the manuscript for surgical content. MB and CG corrected the manuscript. All authors read and approved the final manuscript.
